# Platinum Nanoparticles Decrease Reactive Oxygen Species and Modulate Gene Expression without Alteration of Immune Responses in THP-1 Monocytes

**DOI:** 10.3390/nano8060392

**Published:** 2018-06-01

**Authors:** Francesca Gatto, Mauro Moglianetti, Pier Paolo Pompa, Giuseppe Bardi

**Affiliations:** 1 Istituto Italiano di Tecnologia, Nanobiointeractions & Nanodiagnostics, Via Morego 30, 16163 Genova, Italy; francesca.gatto@iit.it (F.G.); mauro.moglianetti@iit.it (M.M.); pierpaolo.pompa@iit.it (P.P.P.); 2 Istituto Italiano di Tecnologia, Nanobiointeractions & Nanodiagnostics, Center for Bio-Molecular Nanotechnologies, Via Barsanti Arnesano, 73010 Lecce, Italy

**Keywords:** platinum nanoparticles, monocytes, cytokines, chemokine receptors, inflammation

## Abstract

Platinum nanoparticles (PtNPs) attract great attention due to their efficient catalysis and good degree of cytocompatibility, but information about their effects on the human immune system is still missing. Monocytes are key cells of the innate immune system and the understanding of their reactions to PtNPs is crucial in view of any feasible application to human pathologies. Here, we evaluate the internalization of citrate-coated PtNPs into THP-1 monocytes and its consequences on immune cell responses. We found that the presence of intracellular PtNPs efficiently reduce reactive oxygen species (ROS) without affecting cell viability. The physiological expression of the immune receptors Cluster of Differentiation 14 (CD14), CD11b, CC-Chemokine Receptor 2 (CCR2) and CCR5 and the expression of cytokines and chemokines are not compromised by the presence of PtNPs within THP-1 cells. On the other hand, the treatment with PtNPs modulates the transcription of sixty genes, some of them involved in lipopolysaccharide (LPS) signaling in different cells. However, the treatment with PtNPs of monocytes does not compromise the LPS-induced increase of cytokines in THP-1 monocytes in vitro. Our results demonstrate that citrate-coated PtNPs are non-toxic, perform efficient intracellular reactive oxygen species (ROS) scavenging activity and possess good immune-compatibility, suggesting them as feasible synthetic enzymes for applications in nanomedicine.

## 1. Introduction

The intrinsic catalytic activity of platinum nanoparticles (PtNPs), as well as other nanomaterials such as fullerenes, palladium and cerium-oxide nanoparticles (NPs), allows efficient reactive oxygen species (ROS) removing inside cells [1,2,3]. Several results demonstrate that PtNPs are promising candidates to develop synthetic enzymes (nanozymes) for applications in health sciences [4,5,6,7,8,9,10,11,12,13]. Pristine PtNPs or PtNPs coated with biocompatible materials have shown good cytocompatibility, although some conflicting results are present in literature [14]. The particle physicochemical properties like surface chemistry, size and shape regulate their biological fate and eventually their potential toxicity [15]. The absence of contaminants (e.g., endotoxin, Pt precursors, toxic unreacted reagents, organic solvents, etc.) during their synthesis process is a crucial point to produce biocompatible colloidal suspensions for medical aims [16]. All these parameters influence the NP delivery and its toxicological profile, often triggering unexpected immune reactions and, hence, increasing the contradiction of some results.

The response of the immune system to PtNPs is poorly investigated. The available data describe an anti-inflammatory activity of PtNPs in murine macrophages previously stimulated with LPS [17]. Blood circulating monocytes, which are the precursors of tissue patrolling macrophages, are key cells of the innate immune system able to discriminate between “self” and “non-self” molecules [18], and aberrantly activated in acute and chronic inflammatory pathologies [19]. At the site of inflammation, cell-to-cell signals, like chemokines (chemo-attractant cytokines), mediate rapid monocyte mobilization binding their cognate receptors on the membrane and contributing to their differentiation into macrophages or dendritic cells that actively phagocytize “non-self” bodies. Furthermore, monocytes regulate growth and differentiation of the other immune cells through the release of specific cytokines, to tailor immune responses against the diverse pathogens [19]. Then, the innate immune reaction to any foreign substance, including PtNPs, is a fundamental step to be assessed in order to validate its biocompatibility. Here we show the interaction and the response of THP-1 monocytes to monodispersed 5 and 20 nm citrate-coated PtNPs. We did not find any PtNP-induced toxicity, despite the evident NP internalization within the cells. Interestingly, we observed PtNP-mediated ROS reduction and discovered their ability to modulate gene transcription without alteration of inflammatory cytokine release. In particular, we demonstrated very low impact of PtNPs on the immune-physiological response and following LPS-mediated monocyte activation.

## 2. Materials and Methods

### 2.1. Platinum Nanoparticles Synthesis and Characterization

Citrate-capped PtNPs were synthetized avoiding potentially dangerous contaminants during the synthetic procedure and characterized by transmission electron microscopy (TEM) using a Jeol JEM 1011 (Jeol, Akishima-shi, Japan) as previously described [9]. Limulus test (Lonza, Basel, Switzerland) was performed to guarantee NP batches being endotoxin-free. PtNPs were analyzed and the size distribution was determined measuring the diameter of at least 500 NPs.

### 2.2. Cell Culture

THP-1 cells (ATCC, Manassas, VA, USA) were grown in RPMI-1640 (Thermo Fisher Scientific, Waltham, MA, USA) supplemented with 10% FBS (Thermo Fisher Scientific, Waltham, MA, USA), 1% Penicillin-Streptomycin (Sigma-Aldrich, Saint Louis, MO, USA) and 0.05 mM 2-mercaptoethanol (Thermo Fisher Scientific, Waltham, MA, USA) in a 5% CO_2_ humidified atmosphere at 37 °C.

### 2.3. Transmission Electron Microscopy (TEM) Analysis of Cellular Internalization of PtNPs

THP-1 cells were incubated with 50 μg/mL PtNPs for 24 h and washed twice with RPMI and fixed for 45 min in a fixative solution (2% Glutaraldehyde in complete culture medium). The samples were centrifuged and the pellet fixed again with 1.5% Glutaraldehyde solution in Na-Cacodylate buffer 0.1 M. A final post-fixation (2 h) in 1% OsO_4_ solution in Na-Cacodylate buffer 0.1 M was performed. The fixed samples were stained overnight in a 1% Uranyl acetate aqueous solution at 4 °C. After several washes in water, samples were completely dehydrated with a scale of Ethanol, transferred in Propylene Oxide and finally infiltrated with epoxy Spurr™ (SPI-Chem) resin. Once the resin has hardened for 48 h in oven at 65 °C, 70 nm thick sections were cut with a Leica EM UC6 ultra-microtome. TEM images were collected with a Jeol JEM 1011 (Jeol, Akishima-shi, Japan) electron microscope (Electron Microscopy Facility—Fondazione Istituto Italiano di Tecnologia, Via Morego 30, 16163 Genova), operating at an acceleration voltage of 100 kV, and recorded with an 11 Mp fiber optical charge-coupled device (CCD) camera (Gatan Orius SC-1000).

### 2.4. Intracellular Uptake of Pt NPs by Flow Cytometry Analysis

THP-1 cells (5 × 10^5^ cells/mL, 12-well plates) were incubated with 50 μg/mL of the appropriate PtNPs. After 2, 6 and 24 h incubation, the cells were washed and resuspended in Phosphate Buffered Saline (PBS) (Lonza, Basel, Switzerland) for the flow cytometry analysis. The effect of PtNPs internalization on cellular side scatter was evaluated by flow cytometry with MACSQuant Analyzer (Miltenyi Biotec, Bergish, Germany) using MACSQuantify software (Miltenyi Biotec, Bergish, Germany).

### 2.5. Annexin-PI Assay

Cell viability was quantified by using Annexin V-PI assay (Miltenyi Biotec, Bergish, Germany) according to the manufacturer’s instructions. In brief, the cells (5 × 10^5^ cells/mL, 12-well plates) were incubated with 50 μg/mL of the proper PtNPs for 2, 6 and 24 h. After the treatments, the cells were washed with 1× Binding Buffer at 300× *g* for 10 min, resuspended in 1× Binding Buffer and incubated with Annexin V-FITC for 15 min in the dark at room temperature. Subsequently, the cells were washed adding 1× Binding Buffer, centrifuged at 300× *g* for 10 min and then resuspended in 1× Binding Buffer. Propidium Iodide (PI) solution was added immediately prior to analysis by flow cytometry with MACSQuant Analyzer (Miltenyi Biotec, Bergish, Germany). The percentage of viable, necrotic or apoptotic cells was evaluated using MACSQuantify software (Miltenyi Biotec, Bergish, Germany). 10% DMSO was used as positive control.

### 2.6. WST-8 Assay

Cell metabolic activity was determined using a WST-8 (2-(2-methoxy-4-nitrophenyl)-3-(4-nitrophenyl)-5-(2,4-disulfophenyl)-2H-tetrazolium, monosodium salt) assay (Sigma-Aldrich, Saint Luis, MO, USA) following the manufacturer’s instructions. Briefly, after 2, 6 and 24 h incubation with PtNPs at the proper concentration the cells were washed twice, resuspended in complete culture medium and seeded in 96-well plates a density of 50,000 cells/100 μL. 10 μL of Cell Counting Reagent WST-8 were added to each well and the plates were incubated in a 5% CO_2_ humidified atmosphere at 37 °C for 2 h. The orange WST-8 formazan product was measured on a Synergy HT (Biotek, Winooski, VT, USA) microplate reader at a wavelength of 460 nm. 10% DMSO was used as positive control.

### 2.7. Receptor Expression

The cells (5 × 10^5^ cells/mL) were incubated with 50 μg/mL Pt-NPs for 2, 6 and 24 h. After incubation, the cells were washed with RPMI at 300× g for 5 min, resuspended in RPMI/0.5% Bovine Serum Albumin (BSA) (Miltenyi Biotec, Bergish, Germany), and incubated with fluorescently labelled antibodies (FITC-conjugated mouse anti-human CD195 (CCR5), Alexa Fluor 647-conjugated mouse anti-human CD192 (CCR2), BD Pharmigen; VioBlue-conjugated mouse anti-human CD14, PE-conjugated mouse anti-human CD11b, Miltenyi Biotec) at the manufacturer’s recommended concentration for 15 min on ice in the dark. The cells were then washed and resuspended in RPMI. Cell-associated fluorescence was analyzed by flow cytometry with MACSQuant Analyzer, gating the living cells based on light forward scattering (FSC) and side scattering (SSC). 100,000 events per sample were acquired.

### 2.8. Inflammatory Cytokine Release

THP-1 cells were incubated with 50 μg/mL PtNPs for 6 h, then washed twice and reseeded in fresh medium. IL-1β, IL-8, MCP-1, MIP-1β, RANTES and TNF-α release were evaluated after 24 h with a Bio-Plex MAGPIX Multiplex Reader (Bio-Rad, Hercules, CA, USA) according to the manufacturer’s instructions. 

The same experiment has been performed with LPS-stimulated THP-1 for 24 h after 6 h administration of 50 μg/mL PtNPs. 100 ng/m LPS have been used as positive control.

### 2.9. DCFDH-DA Assay

THP-1 cells were cultured at a density of 5 × 10^5^ cells/mL in 12-well plates in presence of 50 μg/mL PtNPs for 6 h. The cells were washed with sterile PBS and incubated with 5 μM DCFH-DA (2′,7′-dichlorofluorescein diacetate, Molecular Probes) in PBS for 10 min at 37 °C. The cells were then washed with PBS and the DCF fluorescence intensity was measured with MACSQuant Analyzer (Miltenyi Biotec, Bergish, Germany). 1 mM H_2_O_2_ was used as positive control.

### 2.10. RNA Extraction and Microarray Analysis

5 × 10^5^ cells/mL THP-1 cells were treated with 50 μg/mL of 5 nm PtNPs for 6 h. After the incubation time, the cells were washed twice and the total RNA was extracted with the RNeasy Plus Mini kit (Qiagen, Hilden, Geramny) following the manufacturer’s instructions. The transcriptional profile of THP-1 cells was analyzed by Affymetrix genechip Clariom S Human (provided by Cogentech S.c.a.r.l., Milano, Italy). The results were analyzed by Partek Genomics Suite Software (St. Louis, MO, USA).

### 2.11. Statistical Analysis

All the experiments were performed in triplicate, at least in three independent assays. Statistically significant differences were determined by one-way or two-way Analysis of Variance (ANOVA) analysis followed by Bonferroni’s or Tukey’s post hoc test. A *p* value of <0.05 was considered significant.

## 3. Results

### 3.1. Cytocompatibility of PtNPs with THP-1 Monocytes

**Figure 1:**
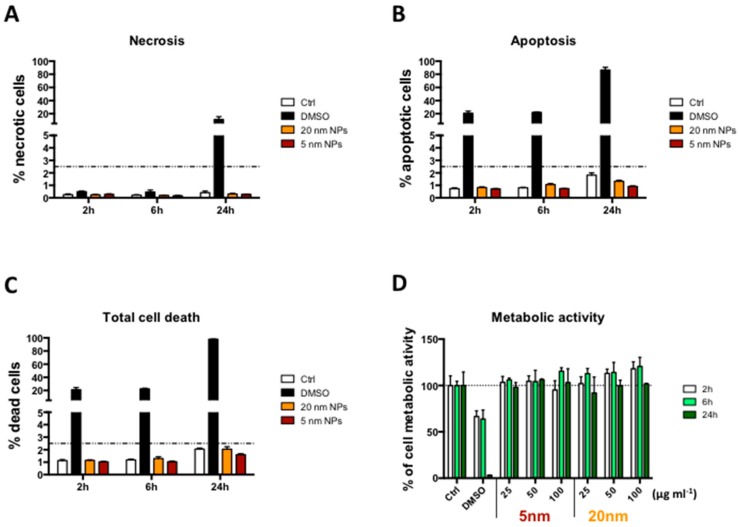
Cell viability. (**A**–**C**) Viability of THP-1 cells after 2, 6 and 24 h exposure to 50 μg/mL PtNPs. Control columns represent untreated cells. Necrotic, apoptotic and total dead cells were evaluated by flow cytometry using the Annexin V/PI assay; (**D**) Cell metabolic activity of THP-1 cells after 2, 6 and 24 h exposure to increasing doses of PtNPs evaluated by WST-8 assay. 10% DMSO was used as positive control in all the experiments. Data are expressed in percentage relative to untreated control cells (empty columns).

5 and 20 nm citrate coated-PtNPs have been synthesized as previously reported [9] and characterized by Transmission Electron Microscopy (TEM) analysis (Figure S1A,B). The two sets of PtNPs had low polydispersity and quasi-spherical shape with pronounced external roughness (flower-like appearance), highly increasing their catalytic surfaces. The potential cytotoxicity of PtNPs, has been evaluated by Annexin V/PI and WST-8 assays performed on NP-treated THP-1 cells. 5 and 20 nm PtNPs did not induce significant necrotic or apoptotic cell death (Figure 1A–C), neither decrease THP-1 metabolism up to 100 µg/mL within 24 h (Figure 1D).

### 3.2. THP-1 Monocyte Internalization of PtNPs

**Figure 2:**
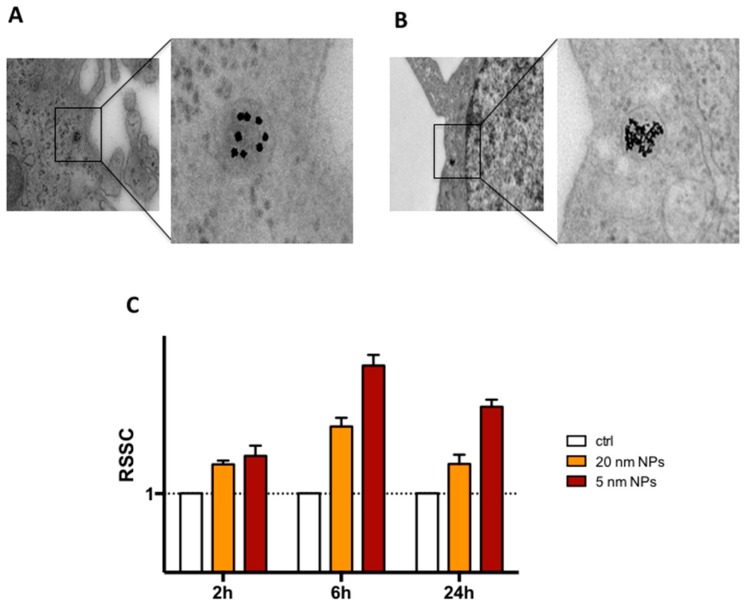
Cellular uptake and intracellular localization of PtNPs. High magnification TEM images of internalized 20 nm (**A**) and 5 nm (**B**) 50 μg/mL PtNPs in THP-1 cells; (**C**) Time variation of Side Scatter in THP-1 cells treated with 50 μg/mL PtNPs evaluated by flow cytometry. Data are normalized to untreated control (empty columns).

The administration of PtNPs to THP-1 monocytes cultured in complete RPMI medium resulted in cell internalization of the NPs, as demonstrated by TEM (Figure 2A,B). High magnification intracellular TEM images highlight the endosomal localization of the particles and the higher amount of 5 nm PtNPs in the subcellular compartment compared to the 20 nm. Experimental data show that maximal internalization of NPs is reached after 6 h, as confirmed by increased light side scattering in flow cytometry (Figure 2C) and by elemental analysis using Inductively Coupled Plasma Atomic Emission Spectrometer (ICP-AES) (Figure S2). The slight decrease after 24 h could be possibly due to exocytosis mechanisms for endocytosed NPs [20]. We did not analyze further time points in our experiments since we studied the consequences of acute administration of PtNPs to THP-1 cells, which complete their cell cycle after one day in culture and double in 35–50 h [21].

### 3.3. Immune Receptor Expression in the Presence of PtNPs

**Figure 3:**
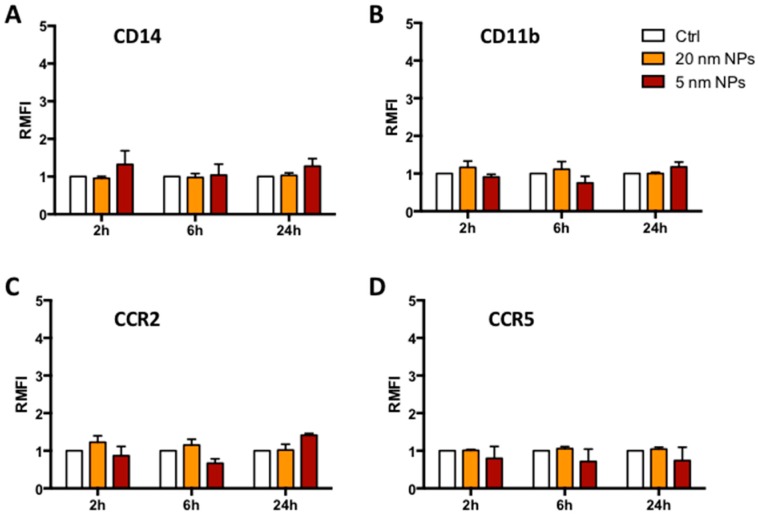
THP-1 receptor expression. Time dependent relative median fluorescence intensity (RMFI) evaluated by flow cytometry of CD14, CD11b, CCR2 and CCR5 after 50 μg/mL Pt-NP treatment of THP-1 cell. Control (Ctrl, empty columns) represents untreated cells.

Modulation of immune receptor expression on the cell surface is an indication of possible membrane alteration or inappropriate response to intercellular communication. As PtNPs interact with monocyte membranes to proceed with their internalization, we evaluated by flow cytometry (Figure 3) the expression of the LPS co-receptor CD14 and the CD11b, whose up-regulation in different leukocytes is considered a marker of innate immune response [22]. We also analyzed two inflammatory chemokine receptors, namely CCR2 and CCR5, usually driving monocyte recruitment to the sites of inflammation [23]. The presence of 5 or 20 nm citrate-PtNPs for 24 h did not induce any significant variation in the receptor expression on the cell surface, suggesting low impact of PtNPs on membrane receptor presentation.

### 3.4. Cytokine Expression in the Presence of PTNPs

**Figure 4:**
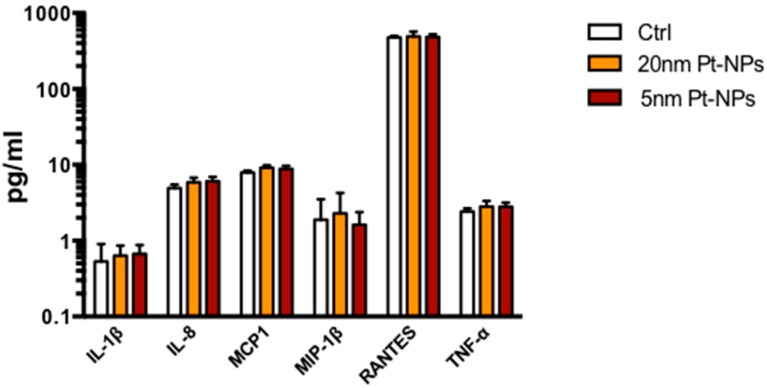
Cytokine release. Column graphs show IL-1β, IL-8, MCP-1, MIP-1β, RANTES and TNF-α levels released by THP-1 24 h after treatment with 50 μg/mL PtNPs for 6 h, measured by Bio-Plex MAGPIX Multiplex Reader. Control columns (Ctrl, empty columns) represent untreated cells. Results are expressed in pg/mL in logarithmic scale.

Although we did not find signs of toxicity in NP-treated THP-1 monocytes, we investigated whether the presence of PtNPs could affect other cellular mechanisms involved in the immune response. Since monocytes coordinate immunity by the release of cytokines as mediators of inter-cellular communication, we treated THP-1 with 50 μg/mL NPs for 6 h, previously demonstrated as the highest time point of particle internalization curve in our cell model (Figure 2), and cytokine levels measured 24 h after washing. The extracellular release of IL-1β, IL-8, MCP-1, MIP-1β, RANTES and TNF-α by PtNPs-treated monocytes was not different from untreated cells by both the NPs of different size (Figure 4). To confirm the normal production of cytokines by THP-1 we used LPS stimulation as positive control (Figure S3). This result emphasizes the immune-compatibility of citrate-coated PtNPs with THP-1 in vitro and their unstimulated release of cytokines.

### 3.5. Reactive Oxigen Species ( ROS) Scavenging Activity of PtNPs in THP-1 Monocytes

**Figure 5:**
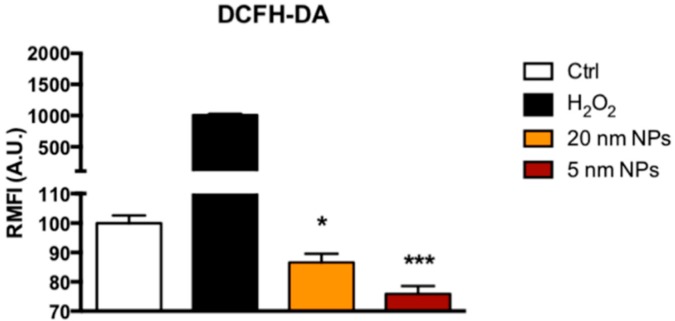
Antioxidant activity. ROS levels in THP-1 cells after 6 h exposure to 50 μg/mL PtNPs evaluated by the DCFH-DA assay in flow cytometry. Control (empty column) represents untreated cells. 1 mM H_2_O_2_ was used as positive control (* *p* < 0.05, *** *p* < 0.001).

Interestingly, we detected statistically significant decrease of ROS in THP-1 cells, measured by DCFH-DA assay in flow cytometry (Figure 5), in the presence of PtNPs vs. untreated cells. 5 nm PtNPs were more efficient in ROS scavenging activity (24% reduction vs. untreated) than 20 nm PtNPs (13% reduction vs. untreated). The higher surface area/mass of the 5 nm NPs and their increased internalization (Figure 2B,C) into the cells might have amplified their efficiency. This result would be in agreement with the improved catalytic reaction by smaller NPs shown by Guarnieri and colleagues with 2.5 nm PtNP [24]. We demonstrated this effect for the first time in monocytes and confirmed what previously observed in other cell types by our group [9,24], and others [17].

### 3.6. Gene Transcription in THP-1 Treated with PtNPs

**Figure 6:**
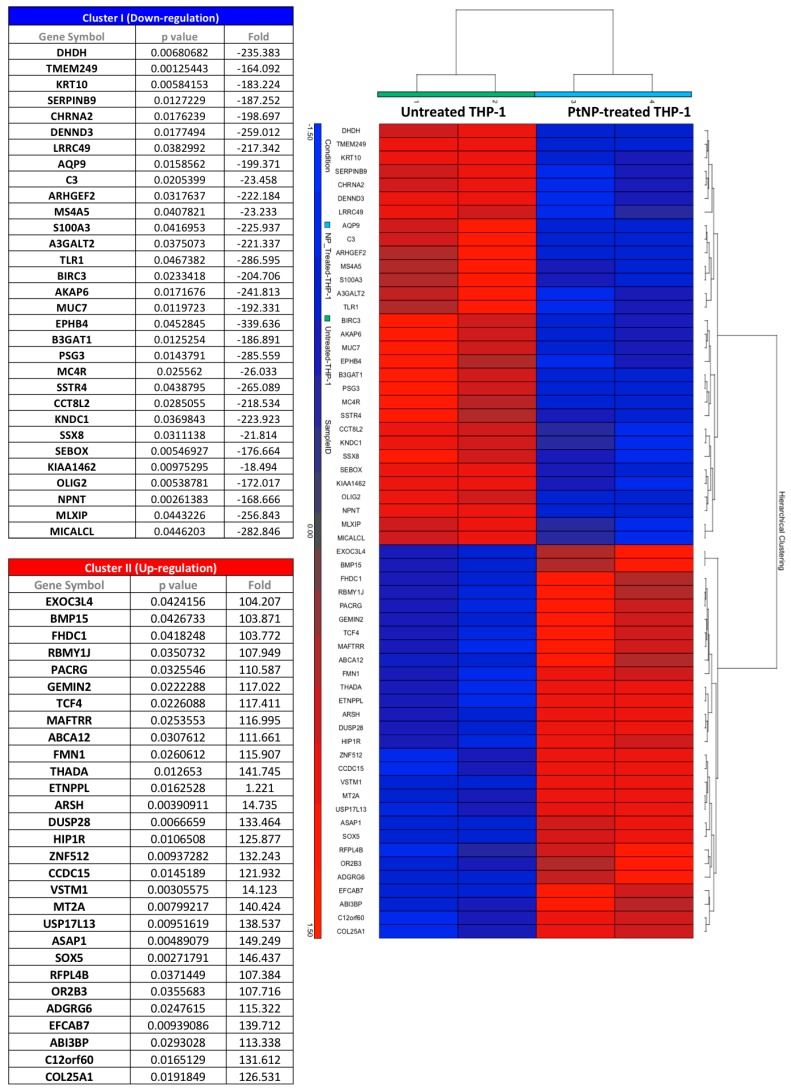
Gene expression—Hierarchical gene clustering. Gene transcription after 6 h 50 μg/mL 5 nm PtNPs treatment evaluated by microarray analysis. The heat map shows significant changes in a group of selected genes due to PtNPs exposure in THP-1 cells. Two clusters were detected, one due to down-regulation of the genes (blue) and other due to up-regulation of the genes (red) in NP-treated vs. NP-untreated cells. Fold change and *p*-value of the genes in the clusters are given on the left.

As consequence of the previous result, we decided to investigate gene transcription by RNA microarray technology in THP-1 treated with the 5 nm PtNPs, which have shown increased internalization (Figure 2B,C) and ROS reduction (Figure 5). Most of the probed genes showed unaltered transcription (Figure S4A), in reasonable agreement with the unaffected cell viability (Figure 1) physiological membrane protein expression (Figure 3) and basal cytokine release (Figure 4). However, among the 20,893 transcripts analyzed with the Affymetrix Human Clariom S array, we identified a group of 60 genes significantly down or up-regulated in the presence of PtNPs (Figure 6 and Figure S4B). Surprisingly, some of the downregulated genes have been related to pathogen dependent inflammatory pathways in lymphatic endothelial cells through caspase-1 (i.e., tlr1 [25], birc3 [26], serpinb9 [27]), somatostatin receptor expression in pulmonary tissues (sstr4 [28]), production of complement components (c3 [29]), neutrophil inflammatory response (aqp9 [30]) and leukemia progression (ephb4 [31]). The PtNP-induced upregulation of vstm1 and asap1 genes involves pathways regulating leukemia cell differentiation [32] and maturation [33]. The PtNP-treatment dependent gene upregulation of mt2a, which is coding for a methallothionein involved in the oxidative stress pathways and LPS induced inflammatory response in mice [34], highlights possible modification of innate immune response by PtNPs. The observed Pt-dependent modification of the transcription of other genes is difficult to relate to known cytokine pathways, since the specific function of their translated proteins is still not, or poorly, known in monocytes with a few indications in other tissues (e.g., sox5 [35], znf512 [36], efcab7 [37], thada [38], kndc1 [39]), albeit a role in innate immune response cannot be excluded.

Gene transcription results indicate feasible interaction of PtNPs with the innate response. However, no significant differences have been observed in resting THP-1 presenting PtNPs in their intracellular compartments (Figure 1, Figure 2, Figure 3 and Figure 4). We previously demonstrated that PtNPs do not affect THP-1 growth and differentiation from monocytes to macrophages [40], restricting the importance of the downregulated genes belonging to these pathways in PtNP-treated monocytes [25,26,27,28,29,30,31,32,33].

### 3.7. Cytokine Expression in PtNP-Treated THP-1 after LPS Stimulation

**Figure 7:**
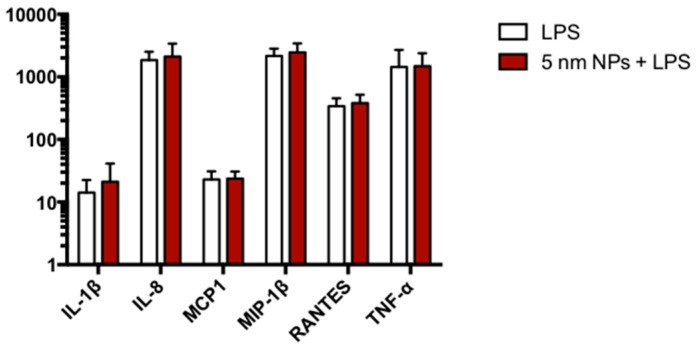
Cytokine release after LPS exposure. Column graphs show IL-1β, IL-8, MCP-1, MIP-1β, RANTES and TNF-α levels released by THP-1 after LPS exposure. The cells were treated for 6 h with 50 μg/mL 5 nm PtNPs, then washed and exposed to 100 ng/mL LPS for 24 h. LPS columns (empty columns) represent untreated THP-1 cells stimulated with LPS.

Gene transcription analysis do not exclude potential incorrect regulation of immune response to pathogenic stimulation in PtNPs-treated monocytes. Since the altered transcription of some genes, like tlr1, c3 and mt2a, is involved in LPS pathways in other cell types, we tested inflammatory cytokine release in LPS-activated THP-1 monocytes 24 h after the administration of 50 µg/mL PtNPs for 6 h. As shown in Figure 7, no differences between NP-treated and untreated monocytes was detected.

## 4. Discussion

The PtNPs have already been demonstrated as ROS scavengers in cell lines and primary cells with pathological oxidative stress conditions [1,9] suggesting a potential role as synthetic nanoparticle-based enzymes (“nanozymes”).

In the present manuscript, we have shown the highly monodispersed citrate-coated PtNPs (Figure S1) are not toxic (Figure 1) and can be internalized by human THP-1 monocytes (Figure 2). The good degree of cytocompatibility up to 100 μg/mL (Figure 1) is supported by our observation that PtNP-treated cells normally express immune receptors like CD14, CD11b, CCR2 and CCR5 indicating that their synthesis and the intracellular trafficking to the plasma membrane is not altered by the presence of PtNPs (Figure 3). As well, the basal release of inflammatory cytokines by resting THP-1 cell line is also not significantly modulated after NP treatment (Figure 4). Cytokine release experiments have been performed to detect the specific role of up-taken particles at the maximum time point of internalization, as indicated by TEM, flow cytometry (Figure 2) and ICP-AES (Figure S2). PtNPs in the extracellular medium can bind proteins, including cytokines, and underestimate their actual amount in the tested supernatants (data not shown).

Although toxicity and immune-responses of monocytes seems not compromised by the presence of PtNPs, we demonstrated an anti-oxidant activity by ROS reduction in the same cells (Figure 5). PtNP interference with fluorescent probes could raise some concerns, as shown for AuNPs and AgNPs [41]. Actually, plasmon resonance of platinum is in the UV region. Its visible spectrum does not have absorbance at 488 wavelength like Au and Ag. The absence of direct interference with DCFH within cells has previously been demonstrated by our group in HeLa cells [9]. In this paper are shown no differences between PtNPs loaded cells and controls. It is important to be mentioned that in THP-1 the basal ROS level is higher than in HeLa cells, in which the decrease of ROS by 5 nm PtNPs can be detected only after H_2_O_2_ treatment. Indeed, the platinum catalytic efficiency is related to the surface/volume ratio as demonstrated by the increased activity of 2.5 nm PtNPs, which are able to reduce the basal ROS in the same cell line [24].

Genetic analysis by RNA microarray technology revealed PtNPs-induced statistically significant alteration of only 60 genes, some of them potentially involved in inflammatory pathways (Figure 6). It is worth to mention that most of the genes are normally transcribed in PtNP-treated THP-1 and perform regular physiological activities, as demonstrated by their viability, the expression of membrane receptors and cytokine production. Anyway, the down- or up-regulation of all the 60 RNA transcripts shown in Figure 6 renders quite difficult the precise role of the PtNPs in the several cell mechanisms. We focused our attention on the PtNP-dependent modulation of trl1 (Toll-Like-Receptor 1), c3 (Complement component 3), birc3 (baculoviral IAP repeat containing 3), sstr4 (Somatostatin receptor 4), serpinb9 (serpin family B member 9), and mt2a (metallothionein 2a) genes which have been shown to participate some inflammatory responses in other cell types following pathogenic stimuli like LPS [25,26,27,28,29,34]. So, we activated monocytes with LPS either in the presence or absence of PtNPs and measured the cytokine release. Our data do not show differences in the LPS-induced increase of IL-1β, IL-8, MCP1, MIP-1β, RANTES and TNF-α (Figure 7) between PtNP-treated and control THP-1 monocytes. Intriguingly, Rehman and colleagues [17] observed an anti-inflammatory effect of pectin-coated 2.5 nm PtNPs in LPS-induced inflammatory pathways using RAW264.7 murine macrophages. These results underline how slightly different PtNPs and the differentiation state of the monocyte/macrophage lineage may play an important role in the detected immune responses [17,40]. The complex signaling that regulate immunity could be also coordinated by genes with multiple functions. In the present study, the transcription of other genes involved in cell maturation and leukemia progression, such as ephb4, aqp9, asap1, vstm1 was also altered by PtNPs [30,31,32,33]. Nevertheless, we have previously demonstrated that citrate-coated PtNPs do not induce major effects in the differentiation process of THP-1 macrophages [40]. However, a more complex cascade-like mechanism cannot be excluded. Further research will be required to understand if the blockade of just one, or a few, of these genes could subsequently impair the transcription of other genes involved in immune mechanisms.

## 5. Conclusions

We have shown that citrate-coated PtNPs are not cytotoxic and immune-compatible with THP-1 monocytes. We measured PtNP ROS scavenging activity, likely contributing to the observed modulation of 60 genes. Finally, we demonstrated that PtNPs do not affect LPS-induced inflammatory cytokine upregulation and excluding the role of modulated genes in this phenomenon.

## Supplementary Materials

The following are available online at http://www.mdpi.com/2079-4991/8/6/392/s1, Figure S1: TEM images of PtNPs of 20 nm (A) and 5 nm (B) diameter with relative NPs size distribution analysis. Figure S2: Representative quantification of internalized PtNPs/cell by ICP-AES. The control column represents untreated cells. Figure S3: Cytokines release after LPS 100 ng/ml used as positive control. Figure S4: Scatter plot of untreated vs PtNPs treated THP-1 cells (A) and volcano plot of the 60 modulated genes (B).
