# Supplementary material for: Platinum Nanoparticles Decrease Reactive Oxygen Species and Modulate Gene Expression without Alteration of Immune Responses in THP-1 Monocytes
Source: Nanomaterials (Basel). 2018 Jun 1;8(6):392. doi: 10.3390/nano8060392 (PMC6027382; doi:10.3390/nano8060392)
Supplement: Supplementary file 1 [file nanomaterials-08-00392-s001.pdf]

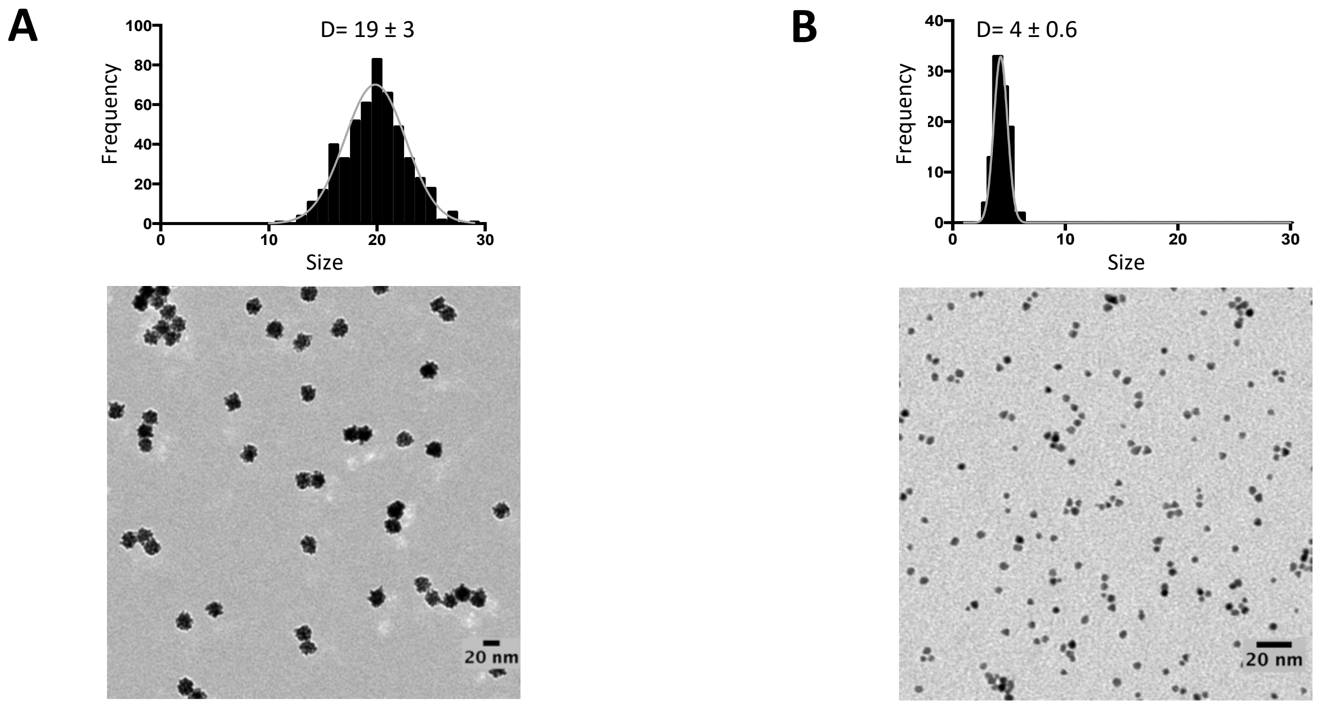

Supplementary Figure 1

TEM images of PtNPs of 20 nm (A) and 5 nm (B) diameter with relative NPs size distribution analysis.

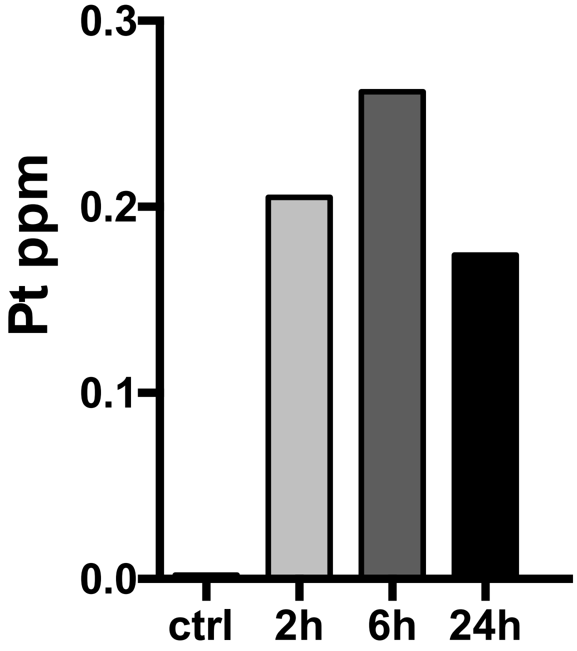

Supplementary Figure 2
Representative quantification of internalized PtNPs/cell by ICP-AES. The control column represents untreated cells.

Methods

Quantification of intracellular uptake of PtNPs by elemental analysis
1 x 10^7 cells per well in 6-well plates were incubated with 50 μg/ml PtNPs for 2, 6 and 24h. After treatments, cells were washed twice in sterile PBS and digested overnight with 1 ml of aqua regia. Then, the samples were diluted to 10 ml with MilliQ water and the amount of internalized Pt-NPs per cell was directly quantified by Inductively Coupled Plasma Atomic Emission Spectroscopy (ICP-AES) (Agilent 720/730 spectrometer).

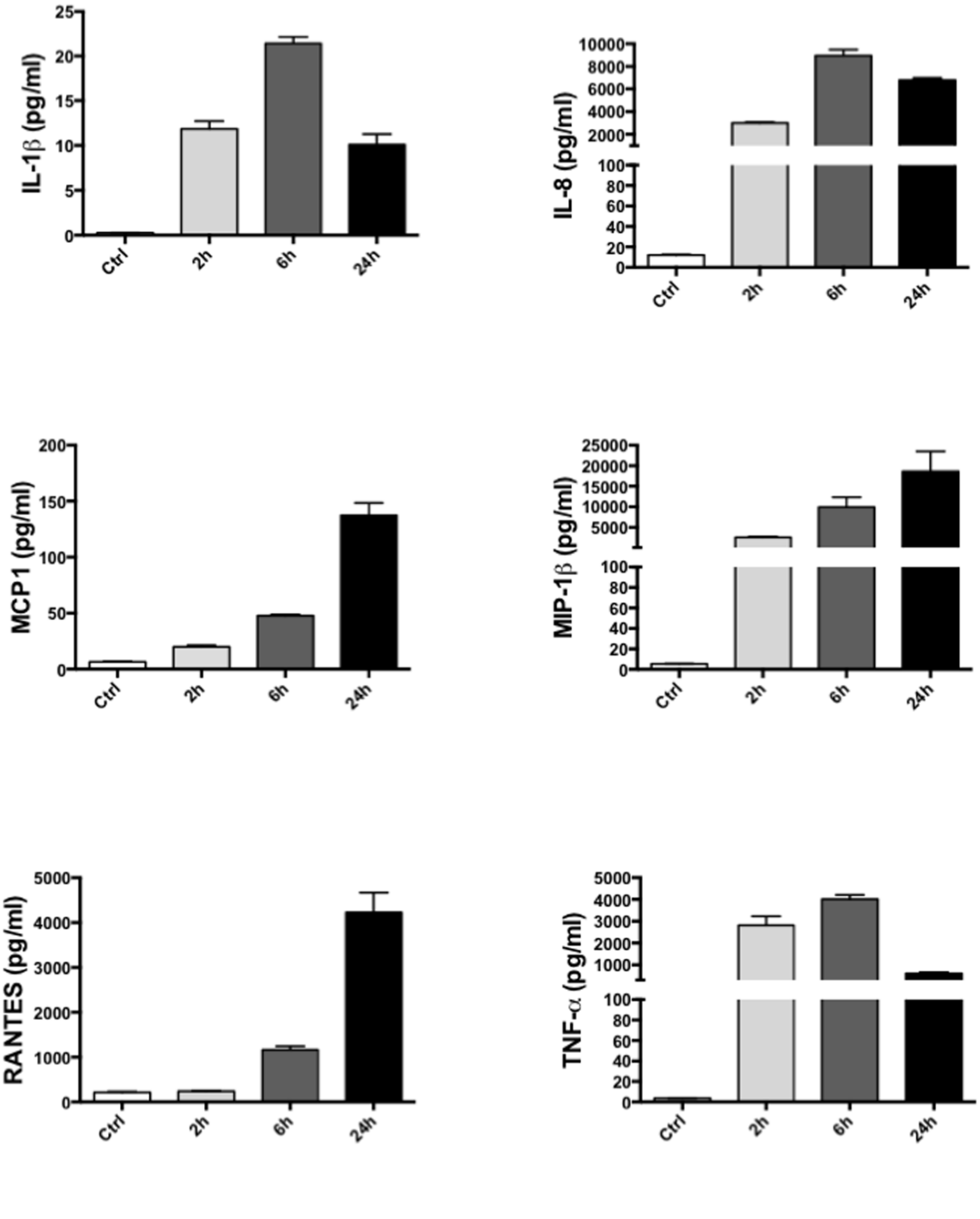

Supplementary Figure 3

Cytokines release after LPS (Lipopolysaccharide) 100ng/ml used as positive control

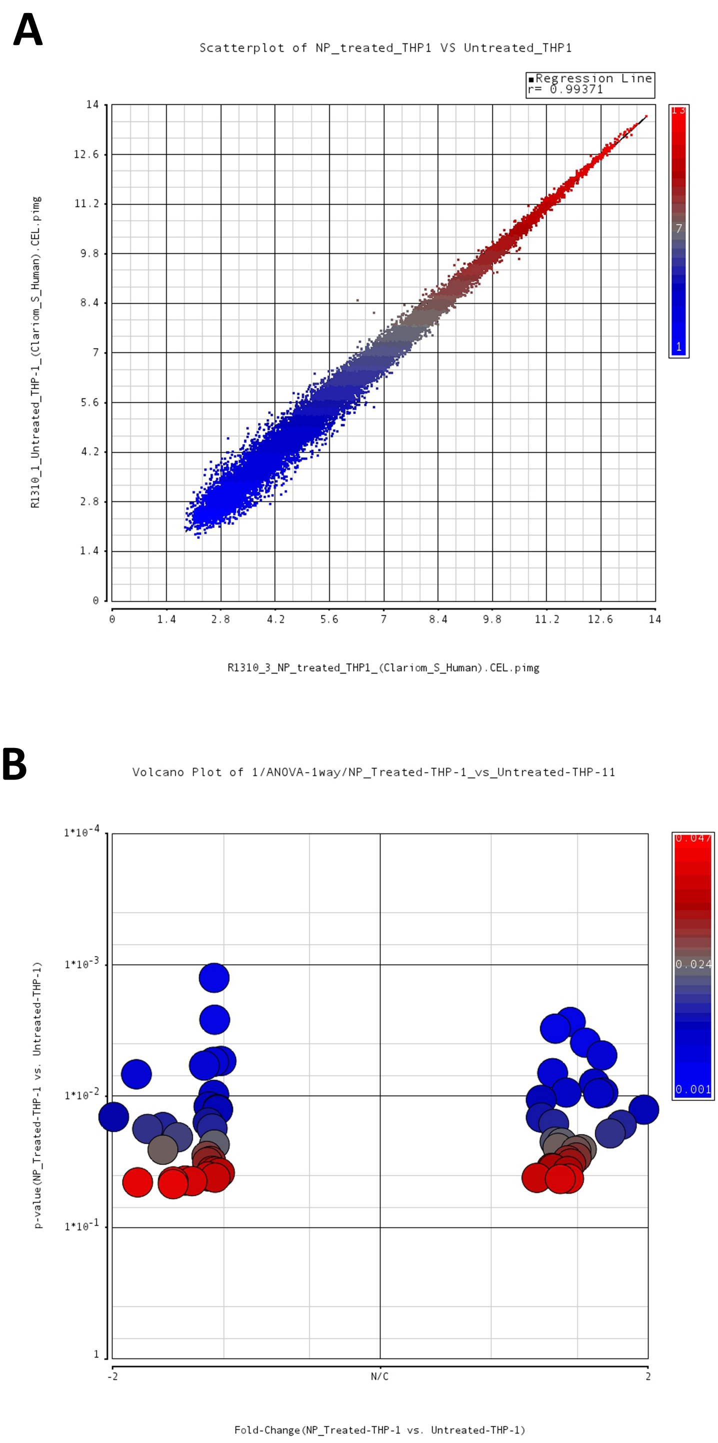

Supplementary Figure 4

Scatter plot of untreated vs PtNPs treated THP-1 cells (A) and volcano plot of the 60 modulated genes (B).
